# Study on Microstructure and Properties of Micron Copper Powder-Liquid Metal Gallium Composite Interconnect Joint

**DOI:** 10.3390/ma19020314

**Published:** 2026-01-13

**Authors:** Bo Wang, Siliang He, Guopei Zhang, Menghao Liu, Kaixuan He, Wei Huang, Kailin Pan

**Affiliations:** 1 East China Institute of Photo-Electron IC, Bengbu 233000, China; wangbo_guet@163.com (B.W.); hekxsr@163.com (K.H.); 2 Guangxi Key Laboratory of Advanced Packaging and System Integration, School of Mechanical and Electrical Engineering, Guilin University of Electronic Technology, Guilin 541004, China; siliang_he@guet.edu.cn (S.H.); huang0773@guet.edu.cn (W.H.); 3 Research Institute of Sun Yat-sen University in Huizhou, Huizhou 516081, China; 4 Goertek Inc., Weifang 261000, China; 0521825@163.com

**Keywords:** liquid gallium, transient liquid phase bonding, intermetallic compound, shear strength, electrical conductivity

## Abstract

Liquid gallium (Ga) enables low-temperature transient liquid phase bonding (TLPB), but optimizing microstructure and joint performance remains challenging. Here, we developed a copper (Cu)-powder/liquid-Ga composite paste for Cu/Cu interconnects and systematically studied the effects on the interconnect joint performance of Cu powder particle size (Cu_PS_, 10–20, 20–30 and 30–40 μm) and Cu mass fraction (Cu_MF_, 10–30 wt%). The microstructure, electrical conductivity, and shear strength of the joint were evaluated, followed by an assessment of bonding temperature, pressure, and time. Under bonding conditions of 220 °C, 5 MPa and 720 min, a dense intermetallic compound (IMC) microstructure predominantly composed of Cu_9_Ga_4_ and CuGa_2_ was formed, yielding an electrical conductivity of approximately 1.1 × 10^7^  S·m^−1^ and a shear strength of 52.2 MPa, thereby achieving a synergistic optimization of electrical and mechanical properties; even under rapid bonding conditions of 220 °C, 5 MPa and 1 min, the joint still attained a shear strength of 39.2 MPa, demonstrating the potential of this process for high-efficiency, short-time interconnection applications. These results show that adjusting the composite paste formulation and dosage enables Cu–Ga TLPB joints that combine high conductivity with robust mechanical integrity for advanced packaging.

## 1. Introduction

As microelectronic devices advance toward higher performance, integration density, three-dimensional (3D) architectures and power-handling capability [1,2,3,4], increasingly stringent requirements are imposed on packaging interconnect technologies, including low-temperature processing, high reliability, superior thermal management, and compatibility with heterogeneous materials [5,6,7]. Conventional Sn-based solders increasingly fail to meet the requirements of advanced packaging—particularly for power devices, 3D integrated circuits and flexible electronics—because of their limited melting points, susceptibility to electromigration, and the formation of coarse intermetallic compound (IMC) and Kirkendall voids under high current densities [8,9,10,11]. Against this backdrop, gallium (Ga) and its alloys provide promising material solutions to these challenges due to their unique physicochemical properties. Specifically, the low melting point (29.8 °C) of Ga [12] enables low-temperature interconnect processing, reducing thermal damage, and ensuring compatibility with heat-sensitive materials. At the same time, its high boiling point and excellent thermal conductivity facilitate thermal management under high-temperature operating conditions [13,14,15]. Furthermore, Ga reacts with various metals to form high-melting-point IMCs [16,17,18,19,20], thereby maintaining joint structural stability at elevated temperatures even after low-temperature processing. Therefore, these properties allow Ga-based materials to achieve a favorable balance between low-temperature fabrication and high-temperature operation, making them an emerging focus in advanced packaging interconnect research.

Solid-liquid interdiffusion (SLID) and transient liquid phase bonding (TLPB) are two key technologies that exploit low-melting-point metals to produce joints stable at high temperatures [21,22,23,24]. Ga-based alloys offer distinct advantages in both approaches: through low-temperature interfacial reactions with metals such as copper (Cu), nickel (Ni) and gold (Au), they form high-melting-point IMC such as CuGa_2_, Cu_9_Ga_4_ [25], and Ga_7_Ni_3_ [19], thereby realizing the concept of “low-temperature processing for high-temperature use”. Lin et al. [26] introduced Ga-X alloy structures into the Cu/Ga bonding system and systematically investigated interfacial reactions and microstructural evolution during curing, annealing and reflow. Compared with pure Ga, the Ga-X alloy structure effectively suppressed interfacial pit formation and improved bonding reliability. Huang et al. [27] employed a custom-formulated Ga-based solder paste in combination with Ni under-bump metallization (UBM) to obtain an IMC-free fcc-(Ni, Cu, Ga) solid-solution joint. This joint exhibited excellent thermal stability but provided a shear strength of only 1.3 MPa. Lin et al. [25] examined the reaction behaviour of a Cu/Ga/Cu sandwich bicomponent system at 160 °C and observed crack formation in the CuGa_2_ phase region. The study concluded that the brittle CuGa_2_ phase is unsuitable for Ga-based TLPB bonding. Furthermore, Lin et al. [19,28] investigated the reaction mechanism leading to fcc-(Ni, Cu, Ga) solid-solution joints in a Cu/Ni/Ga/Ni/Cu system and achieved a maximum shear strength of 43.5 MPa at a Ni UBM thickness of 100 nm. Chen et al. [29] carried out an in-depth investigation of the interfacial IMC structure, mechanical properties, and void-formation mechanisms in Ga-Cu TLPB joints over the temperature range 150–220 °C. A shear strength of 23.8 MPa was obtained at 220 °C with a holding time of 90 min. Liu et al. [30] examined the interfacial reactions between the liquid Ga and Cu-xNi (x = 0, 2, 6, 10, 14) substrates at room temperature and further evaluated the lap-shear strength of the resulting joints. After 7 days of annealing, a shear strength of approximately 10 MPa was achieved. Froemel et al. [31] examined bonding parameters for low-temperature wafer-level semiconductor substrate joining and their impact on bond–interface properties. In Cu/Ga bonding, the phase transition from CuGa_2_ to Cu_9_Ga_4_ was identified as the primary factor responsible for strengthening the joint, and a shear strength of 90 MPa was obtained after 80 h at 90 °C under these conditions.

However, Ga-based alloys still face several challenges in practical applications. First, their high surface tension and susceptibility to oxidation lead to poor wettability, necessitating treatments such as dilute acid etching [29,32], alloying with elements such as In or Sn [33,34], controlled atmospheres [32,34,35,36,37], or UBM (for example, Au, Pd, Ni or Pt) [20,33,38,39] to improve interfacial contact. Second, brittle IMCs such as CuGa_2_ readily form during interfacial reactions, together with defects such as Kirkendall voids and microcracks [25,40,41], thereby degrading joint mechanical properties and long-term reliability. In addition, the formation of Cu–Ga joints typically requires prolonged bonding or holding time. These requirements substantially prolong production cycles and limit the efficiency of Cu–Ga bonding for large-scale engineering applications. To overcome these bottlenecks, researchers have begun to explore Ga-based composite interconnect materials. By introducing solid metal fillers into liquid Ga to form a “solid liquid” composite system, it becomes possible to actively control interfacial reaction pathways and microstructure. For example, Temnykh et al [16] developed a composite Ga-based solder paste for low-temperature diffusion brazing of metal–ceramic interfaces. This paste consists of a low-melting-point Ga matrix (35–50%) and high-melting-point metallic filler powders. The study examined the effects of filler content, particle size and brazing pressure on the shear and tensile strengths of the joints. Mu et al [42] successfully realized SLID bonding at 2 MPa and 100 °C for 30 h using a composite interlayer of Cu mesh and Ga/Cu solder paste, obtaining a shear strength of 7.908 MPa and a joint thermal contact conductivity as high as 88,315 W·m^−2^·K^−1^. Sommadossi et al [43] used a metallic paste composed of liquid Ga mixed with Al and Ni powders (average particle size 10 μm) to form a multilayer connection zone approximately 50 μm thick between the Cu and Ni substrates, with minimal defects such as voids or cracks. Collectively, these studies demonstrate that composite formation is an effective strategy for enhancing both the performance and the processability of Ga-based joints.

Despite some progress in recent years, a systematic understanding of Ga-based composite interconnects remains limited. The diffusion of metal powders in liquid Ga, their competing reactions with the substrate, and the effect on joint performance microstructure remain unclear. A key challenge is how to promote the formation of high-strength and high-toughness composite interfaces while suppressing the continuous growth of brittle IMCs by optimizing sizes and the content of powder and reaction conditions. In this study, the TLPB behaviour of micron-sized Cu-powder/liquid-Ga composites was comprehensively investigated. By systematically screening different Cu powder particle sizes (Cu_PS_) and Cu mass fractions (Cu_MF_) in the paste, detailed characterization and analysis of microstructure, electrical conductivity, and shear strength of joint were performed using scanning electron microscopy (SEM), energy-dispersive spectroscopy (EDS), four-probe measurements, and shear tests. The effects of bonding temperature, pressure, and duration on microstructure evolution, IMC growth, and joint shear properties were further investigated. These findings provide new insight into the microstructure and performance of Cu–Ga composites during low-temperature bonding and an important theoretical basis for the wider application of Ga-based alloys in microelectronic packaging.

## 2. Materials and Methods

### 2.1. Preparation Methods of Cu–Ga Composite Paste and Joint

The raw materials used in this study were irregular high-purity Cu powder (99.999%) and high-purity Ga metal (99.9999%). To remove surface oxides and organic contaminants, the Cu powder was first ultrasonically cleaned in a 10 wt% citric acid solution for 10 min (40 kHz, 180 W). It was then rinsed three times with deionized water and vacuum-dried at 110 °C for 30 min. The dried Cu powder was sieved using standard sieves to obtain three narrow Cu_PS_ of 10–20 μm, 20–30 μm, and 30–40 μm.

The Cu–Ga composite paste was prepared by centrifugal mixing. Pre-treated Cu powder and liquid Ga were weighed to obtain Cu_MF_ of 10, 15, 20, 25, and 30 wt%, loaded into centrifuge tubes and centrifuged at 2000 rpm for 5 min in a vacuum centrifuge at a specified vacuum level, so that liquid Ga was uniformly coated on the Cu powder surfaces. Subsequently, the paste underwent ultrasonic degassing for 1 min, yielding a silvery-white, Cu–Ga composite paste. To reduce the rate of interdiffusion between Cu and Ga, the Cu–Ga composite paste was stored at −20 °C until use [32].

**Figure 1:**
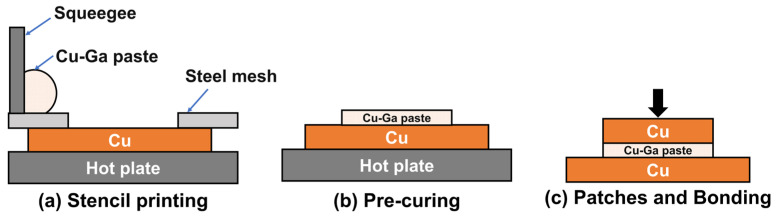
Preparation of interconnect joints based on Cu–Ga composite paste.

Interconnect joints were fabricated by screen printing followed by TLPB. Oxygen-free Cu substrates (3 × 3 × 1 mm^3^ and 5 × 5 × 1 mm^3^, used as the upper and lower substrates, respectively) were ground with 5000-grit sandpaper, polished using 50 nm Al_2_O_3_ suspension, and sequentially cleaned with citric acid, deionized water, and anhydrous ethanol. Figure 1 shows the fabrication process for interconnect joints using Cu–Ga composite paste. To ensure sufficient flowability of the Cu–Ga composite paste, screen printing was carried out on a 45 °C heated platform. The paste was printed onto the Cu substrates using a stainless-steel mesh stencil and then laminated with a second Cu substrate to form sandwich samples. The paste was deposited by manual stencil printing using a stainless-steel stencil (thickness: 120 μm; aperture size: 3 × 3 mm). A hand-held squeegee was used at an angle of approximately 40° to spread the paste through the stencil openings. The sandwich samples were placed in a vacuum heating furnace and heated at 7 °C·min^−1^ to the target temperature (100, 140, 180, 220 or 260 °C), subjected to a uniaxial pressure of 0, 1, 5, or 10 MPa and held at the target temperature for 1, 10, 60, 180, or 720 min. After bonding, the assemblies were furnace-cooled to below 50 °C before the pressure was released. This process promotes interdiffusion reactions between Cu particles and Ga, forming stable Cu–Ga intermetallic phases and sound metallurgical bonding interfaces. Field-emission SEM (TESCAN MIRA LMS) (TESCAN, Brno, Czech Republic) operated in secondary electron (SE) imaging mode and equipped with an EDS (Oxford Xplore 30) (Oxford Instruments, High Wycombe, UK) was used for microstructural observation and phase-composition analysis of ground and polished samples.

### 2.2. Conductivity Testing Method

**Figure 2:**
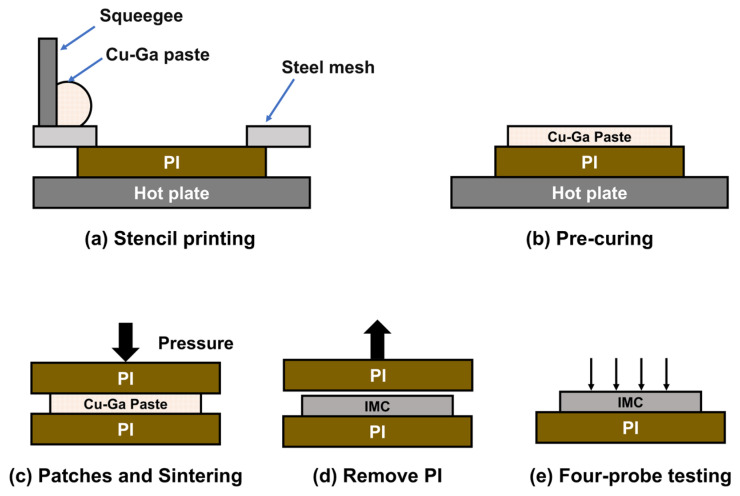
Preparation of conductivity test samples based on Cu–Ga composite paste.

For conductivity measurements, the Cu substrates in the interconnect joints were replaced by polyimide (PI) films. PI exhibits excellent high-temperature resistance and does not participate in the reaction between Cu and Ga [44]. As shown in Figure 2a, Cu–Ga composite paste was screen-printed onto the PI surface using a stainless-steel mesh, forming the sandwich structure illustrated in Figure 2c. Finally, one side of the PI film was removed, and the electrical properties were evaluated using a four-probe test.

Because the PI substrates at both ends do not participate in the reaction, the joint structure of specimens prepared by this method differs from that of Cu/Cu–Ga/Cu interconnects or conventional Cu joints. Nevertheless, we believe that such samples adequately represent the intrinsic electrical properties of Cu–Ga interconnects. The resistivity was measured using the four-probe method with an RTS-9 dual-electrode four-probe tester (Guangzhou Four-Probe Instrument Co., Ltd., Guangzhou, China) and calculated according to Equation (1) [45]:

(1) $$\rho =\frac{V}{I}\omega C$$

where *ρ* denotes electrical resistivity, *V* is the measured voltage, *I* is the applied current, *ω* is the specimen thickness, and *C* is the geometric correction factor; the electrical conductivity *σ* was obtained as *σ* = 1/*ρ*.

### 2.3. Mechanical Testing Methods

In addition to electrical performance, interconnect joints are required to maintain sufficient mechanical integrity. Therefore, shear strength tests were performed to evaluate the robustness of the joints and to correlate joint reliability with microstructural evolution and defects. The shear performance of the sandwich joints was characterized after TLPB. Shear strength tests were carried out at room temperature using an MF1200F (Rui Yin Precision Technology Co., Ltd., Shenzhen, China) testing system at a crosshead speed of 10 μm·s^−1^. The fracture surfaces after shear loading were examined using a 3D laser measuring microscope (OLS4100) (Olympus, Tokyo, Japan) to identify the failure modes.

## 3. Results and Discussion

### 3.1. Microstructural Interfaces and Electrical/Mechanical Properties of Joints with Different Cu_PS_ and Cu_MF_

#### 3.1.1. IMC Phase Identification

**Figure 3:**
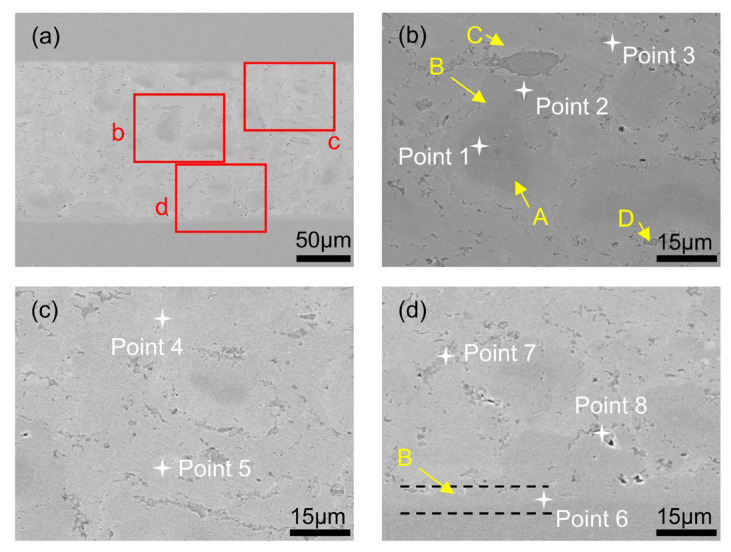
(**a**) SEM images of the joint prepared using a Cu–Ga paste (Cu_PS_ = 30–40 μm, Cu_MF_ = 25 wt%) after reaction at 220 °C and 5 MPa for 12 h; (**b**) enlarged view of rectangle b in (**a**); (**c**) enlarged view of rectangle c in (**a**); (**d**) enlarged view of rectangle d in (**a**). The SEM morphology and corresponding Cu/Ga elemental maps of the joint are shown in Supplementary File, Figure S1.

Figure 3 shows the microstructure of the joint prepared using a Cu–Ga paste (Cu_PS_ = 30–40 μm, Cu_MF_ = 25 wt%) after reaction at 220 °C and 5 MPa for 12 h. The joint consists of four distinct phases: Phase A, a dark phase randomly distributed along the interface; Phase B, a slightly lighter phase that exists independently or coats the surface of Phase A; Phase C, a light phase filling the regions between the other phases; and Phase D, which appears as depressions at the interface in the form of continuous cavities or cracks.

Phase A is a dark phase that is randomly distributed along the joint interface. Based on the EDS point analysis (Table 1), Point 1 shows 99.95 at% Cu and 0.05 at% Ga. This composition strongly suggests that Phase A corresponds to a Cu-rich region, and it is most likely residual Cu, which corresponds to the core of the original Cu powder particles that remained unreacted within the investigated bonding time. The reaction between Cu and Ga initiates at the particle surface and proceeds inward. However, once a Cu–Ga intermetallic layer forms around the particle, further consumption of the Cu core becomes diffusion-limited by Ga transport through the intermetallic shell. As a result, residual Cu cores may persist in the joint microstructure. Phase C appeared light in color and filled the regions between the other phases. Compared with Phase B, it contained less Cu and more Ga. As listed in Table 1, points 3 and 5 contained 65.78 and 66.24 at% Ga and 34.22 and 33.76 at% Cu, respectively. The corresponding Cu/Ga atomic ratios were 0.52 and 0.51, respectively, which were consistent with the stoichiometry of CuGa_2_; Phase C was therefore preliminarily identified as the CuGa_2_ phase. Phase B was located between the Cu substrate (Phase A) and the Ga-rich IMC (Phase C) and formed a continuous interface layer. EDS point analyses of Phase B (Points 2/4/6 in Table 1) show a Cu-rich composition (Cu/Ga ≈ 1.3–1.6). We note that due to the limited spatial resolution and interaction volume of EDS, especially for thin interface layers, the measured composition may be affected by signals from adjacent phases. Considering (i) its interface layer morphology, (ii) the Cu–Ga binary system in which Cu_9_Ga_4_ and CuGa_2_ are the dominant IMCs reported under similar bonding conditions [30,42], Phase B was tentatively assigned as Cu_9_Ga_4_. Finally, Phase D appeared as depressions at the interface in the form of continuous voids or cracks. As shown in Table 1, points 7 and 8 contained 99.18 and 90.27 at% Ga, respectively, and only 0.82 and 9.73 at% Cu. The corresponding Cu/Ga atomic ratios were 0.01 and 0.11, indicating that Phase D was almost entirely composed of Ga and thus represents a Ga-rich phase. The concavities in this region are likely caused by Ga loss during the polishing process. The characteristic appearance of voids or cracks is correlated with the formation of Cu–Ga compounds on the surfaces of the Cu powder. As these compounds form, they progressively consume the residual Ga, leading to the formation of voids or cracks.

To further highlight the relevance of the SEM/EDS-identified IMC, it is worth noting that CuGa_2_ and Cu_9_Ga_4_ differed substantially in composition, crystal structure, and property profiles. CuGa_2_ is a Ga-rich intermetallic crystallizing in a tetragonal structure, whereas Cu_9_Ga_4_ is a Cu-rich intermetallic with a cubic structure [32,46]. First-principles calculations indicate that Cu_9_Ga_4_ is thermodynamically more stable and exhibits higher elastic moduli (bulk/shear/Young’s moduli) than CuGa_2_, suggesting a mechanically stiffer and potentially more load-bearing Cu-rich IMC framework [47]. Therefore, beyond phase identification, the relative fraction and connectivity of Cu_9_Ga_4_ versus CuGa_2_ observed in SEM/EDS are directly relevant to the mechanical integrity and electrical performance of the joints.

#### 3.1.2. The Effect of Cu_PS_ and Cu_MF_ on the Microstructure of Joint

**Figure 4:**
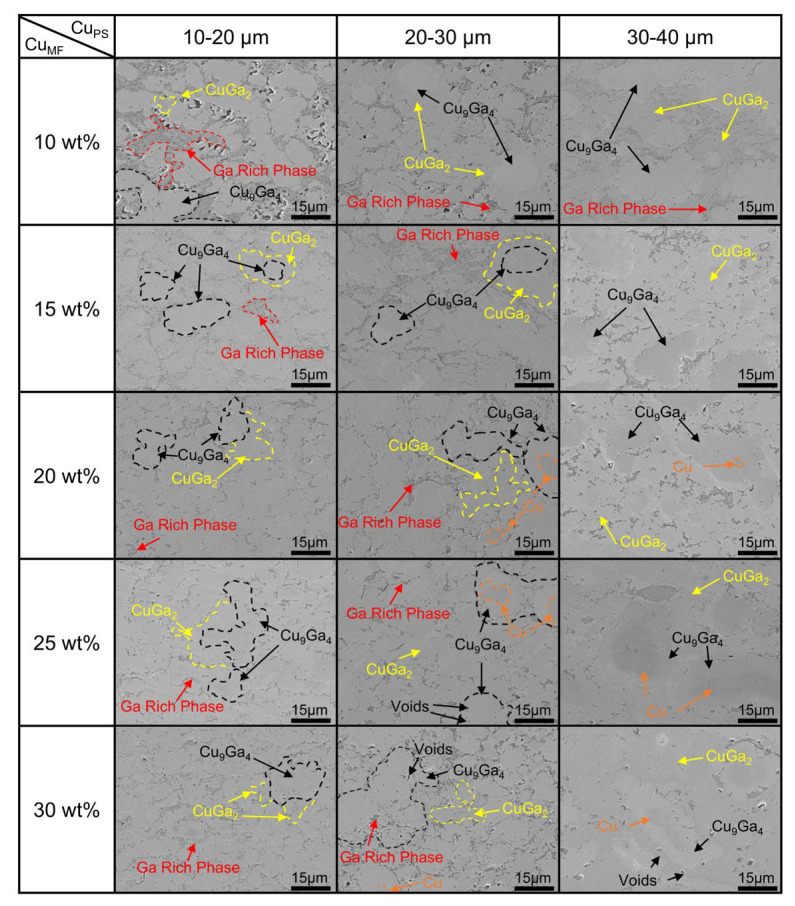
SEM image of a sandwich joint with different Cu_PS_ and Cu_MF_ at 1500× magnification after reacting at 220 °C for 12 h.

Figure 4 presents SEM micrographs of joints prepared with various Cu_PS_ and Cu_MF_. As the Cu_MF_ increases, the joint’s microstructure exhibits a distinct densification trend. At the same Cu_MF_, increasing the Cu_PS_ leads to coarser phase structures within the joint, accompanied by an increase in residual Cu particles and void formation. The results further show that Cu_MF_ and Cu_PS_ significantly affect the microstructure at the joint interface. Under varying Cu_MF_, the phase distribution at the joint interface exhibits systematic changes, reflecting the influence of process parameters on the microstructural characteristics. The corresponding low-magnification (400×) SEM images, which capture the overall joint morphology under different Cu_PS_/Cu_MF_ combinations are shown in Supplementary File, Figure S2.

At a low Cu_MF_, the joint interface exhibits a relatively complex microstructure. Due to the high Ga content, Cu is nearly fully consumed, and the excess Ga forms continuous stripes of a Ga-rich phase throughout the joint. Meanwhile, the Cu_9_Ga_4_ and CuGa_2_ phases predominantly appear in an island-like distribution. This suggests an incomplete reaction between Cu and Ga at low Cu contents, resulting in the formation of a relatively large amount of Ga-rich phase. At the same time, the distribution of Cu_9_Ga_4_ and CuGa_2_ phases within the joint interface becomes more scattered. Such an uneven phase distribution may negatively impact the mechanical properties and electrical conductivity of the joint.

At a high Cu_MF_, the joint interface exhibits a more uniform phase distribution. The proportion of Cu_9_Ga_4_ and CuGa_2_ phases within the joint increases, and these phases are distributed relatively uniformly across the interface. At the same time, the probability of the undiminished Cu phase appearing within the joint is higher. Under these conditions, the presence of Ga-rich phases is reduced, and their distribution shifts to discontinuous regions. This phenomenon can be explained by the fact that, at a higher Cu_MF_, the reaction between Cu and Ga becomes more complete. Once Cu–Ga IMCs (especially Cu_9_Ga_4_ and CuGa_2_) form, they occupy a larger portion of the interface, thereby reducing the formation of Ga-rich phases.

In the joints, the Cu_PS_ significantly influences the morphology and distribution of the Cu_9_Ga_4_ and CuGa_2_ phases. With increasing Cu_PS_, individual Cu_9_Ga_4_ regions become larger, and the CuGa_2_ network formed on the Cu_9_Ga_4_ surface becomes coarser, which reduces the overall microstructural refinement and can decrease joint compactness. As shown in Figure 4, compared with joints prepared using smaller Cu_PS_ (10–20 μm and 20–30 μm), joints prepared using larger Cu_PS_ (30–40 μm) exhibited a coarser IMC framework accompanied by more residual Cu particles. Additionally, voids appeared in certain regions of joints with higher Cu_MF_ (25 wt%, 30 wt%). This phenomenon occurs because after Ga is depleted in localized regions, the IMCs fail to completely fill the joint due to volume contraction effects [41], leading to void formation. At the same time, as Cu_PS_ increases, both the number and volume of voids increase. Finally, the amount of residual Cu powder in the joints increases as the Cu_PS_ increases due to larger particles that have smaller specific surface areas and longer atomic diffusion paths. Consequently, under identical reaction conditions, the reaction rates of joints with a larger Cu_PS_ are slower than those of joints with a smaller Cu_PS_ [48]. This leads to incomplete reaction participation, leaving more residual Cu powder in the joint.

#### 3.1.3. The Effect of Cu_PS_ and Cu_MF_ on the Electrical and Mechanical Properties of Joints

**Figure 5:**
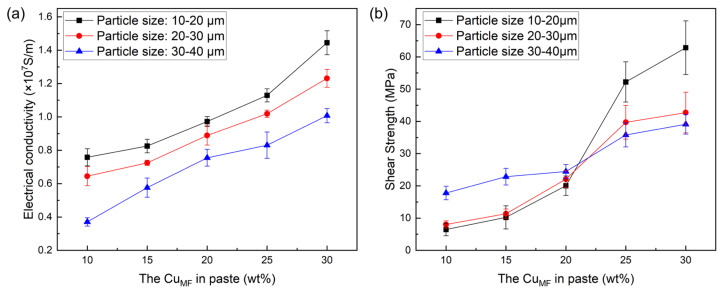
Effect of Cu_PS_ and Cu_MF_ on (**a**) electrical conductivity and (**b**) shear strength of joint.

As shown in Figure 5a, the electrical conductivity of the joints exhibited a clear overall increase with increasing Cu_MF_. All three particle-size ranges followed the same trend, whereas the joints with 10–20 μm Cu_PS_ exhibited the highest conductivity, reaching approximately 1.4 × 10^7^  S·m^−1^  at 30 wt% Cu_MF_. Conversely, joints prepared with 30–40 μm Cu_PS_ exhibited the lowest conductivity. Figure 5b illustrates the influence of Cu_PS_ and Cu_MF_ on the shear strength of the joints. Overall, the shear strength of the joints increased markedly with increasing Cu_MF_ and reached a maximum at 30 wt%. Moreover, the joints with 10–20 μm Cu_PS_ exhibited the highest shear strength (approximately 62.8 MPa) in the high Cu_MF_ range (≥25 wt%), whereas in the low Cu_MF_ range (≤20 wt%), the joints with 30–40 μm Cu_PS_ slightly outperformed those with smaller Cu_PS_. Figure 5 reveals pronounced differences in the electrical and mechanical properties of joints with different Cu_PS_ and Cu_MF_, indicating that Cu powder plays a crucial role in governing microstructural evolution and property optimization.

At a low Cu_MF_ (10–20 wt%), joints prepared with medium-to-small Cu powder (10–30 μm) exhibited a continuous phase dominated by the Ga-rich phase. In joints prepared with large-particle-size Cu powder (30–40 μm), the continuous phase consisted of CuGa_2_. Due to their higher effective specific surface area, which increases the surface free-energy contribution per unit mass [49], fine-particle-size Cu powders (10–20 μm) can react more completely with liquid Ga and form relatively continuous, dense conductive pathways together with the Ga-rich phase, resulting in significantly higher electrical conductivity than in large-particle-size samples. However, the continuous phase dominated by the Ga-rich phase lacks effective load-transfer pathways, resulting in weak interfacial bonding and low overall shear strength of the joint. In contrast, although the large-particle samples (30–40 μm) exhibited lower electrical conductivity, the CuGa_2_ phase-dominated joint structure provided mechanical support at low Ga contents, leading to a higher shear strength. When the Cu_MF_ exceeded 20 wt%, the internal microstructure undergoes a clear transformation, with IMCs becoming the continuous phase under all Cu_PS_ conditions. As the Cu_MF_ increases, the fraction of Ga-rich phases decreases, and a denser IMC network forms that synergistically enhances both the electrical conductivity and shear strength of joints. In contrast, the coarse IMC network formed in joints with larger Cu_PS_ led to an uneven distribution of IMC. This structure hinders uniform stress distribution, promotes localized stress concentrations, and reduces the shear strength of joints. Additionally, the higher void content in joints with larger Cu_PS_ negatively affects both shear strength and electrical conductivity.

**Figure 6:**
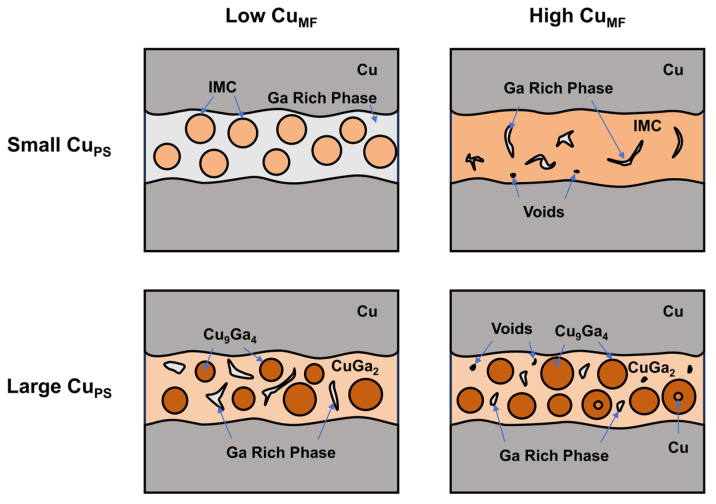
Schematic diagram of joint microstructure at different Cu_PS_ and Cu_MF_.

To better elucidate the influence of Cu_PS_ and Cu_MF_ on the joint microstructure, Figure 6 presents a schematic of the typical microstructure of the Cu powder-liquid Ga joint. In joints with smaller Cu_PS_, the Ga-rich phase envelops dispersed Cu–Ga IMC particles at a low Cu_MF_, whereas the Cu–Ga IMC particles interconnect to form a conductive framework at a high Cu_MF_, with the Ga-rich phase filling the interstices to generate a dense joint structure. As the Cu_MF_ increases, the dominant mechanism shifts from “Ga-rich phase dominated” to “Cu–Ga IMC interconnection dominated”. In joints with a large Cu_PS_, the CuGa_2_ phase covers the surfaces of large Cu_9_Ga_4_ phases and interconnects to form an IMC framework at low Cu_MF_, with the Ga-rich phase distributed as islands within it. At high Cu_MF_, the IMC framework shows no significant change, but the Ga-rich phase shrinks markedly, and fine voids and residual Cu powder appear. Under these conditions, the dominant mechanism in the joint microstructure is “Cu–Ga IMC interconnection dominated”.

### 3.2. Shear Behaviour and Microstructures of Joints Under Varying TLPB Parameters

The Cu–Ga paste composition with a Cu_PS_ of 10–20 μm and a Cu_MF_ of 30 wt% exhibited optimal performance in terms of both electrical conductivity and shear strength. However, due to the high Cu_MF_ in this formulation, the reaction proceeds relatively rapidly, causing the paste to solidify within a short time and thereby adversely affecting the subsequent stencil-printing process. Consequently, the narrow processing window associated with this paste composition is not conducive to large-scale application and industrial implementation. Accordingly, a Cu–Ga paste composition with a Cu_PS_ of 10–20 μm and a Cu_MF_ of 25 wt% was selected for subsequent investigations to optimize the processing window and improve process operability.

**Figure 7:**
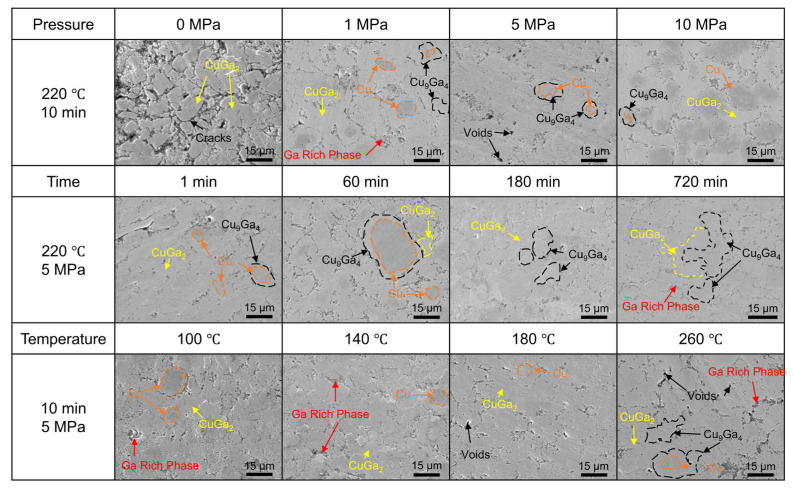
Cross-sectional microstructures of joints fabricated under different TLPB parameters (Cu_PS_ = 10–20 μm; Cu_MF_ = 25 wt%).

Comparative experiments were carried out under different TLPB parameters: (i) bonding at 220 °C for 10 min under pressures of 0, 1, 5 and 10 MPa; (ii) bonding at 220 °C and 5 MPa for bonding times of 1, 10, 60, 180 and 720 min; and (iii) bonding at 5 MPa for 10 min at temperatures of 100, 140, 180, 220 and 260 °C. SEM observations of joint cross-sections revealed the corresponding interfacial microstructures under these different TLPB parameters, as shown in Figure 7. As the bonding pressure increased, defects such as voids and cracks in the joints decreased markedly, and the microstructure became denser. The effect of bonding time on the joint microstructure was manifested primarily in changes in phase composition. With increasing bonding time, Cu and Ga in the joints were continuously consumed, and the volume fraction of IMC gradually increased. Among these IMCs, the fraction of the Cu_9_Ga_4_ phase increased progressively. Within the bonding temperature range of 100–220 °C, the residual Ga-rich phase in the joints decreased with increasing temperature and the microstructure gradually became denser. However, at 260 °C, the Ga-rich phase exhibited an anomalous increase, accompanied by significant additional formation of the Cu_9_Ga_4_ phase. Representative interfacial microstructures under these various pressure, time, and temperature conditions are further provided in Supplementary File, Figure S3.

#### 3.2.1. Effect of Bonding Pressure on Shear Strength and Microstructures of Joints

**Figure 8:**
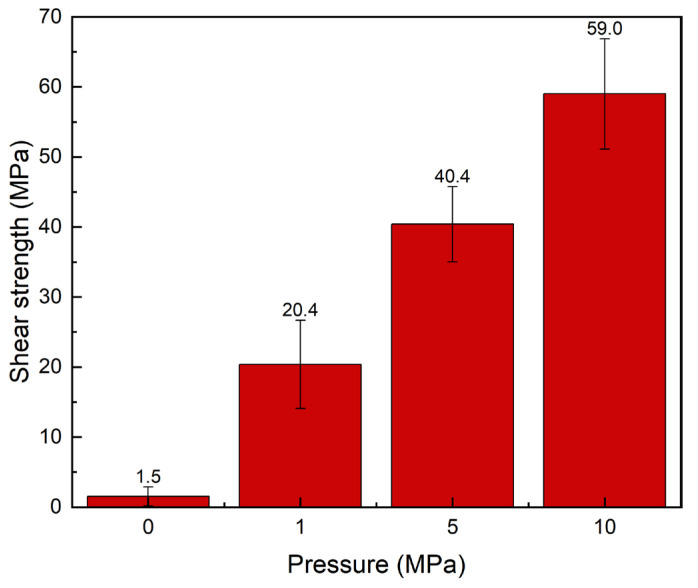
Shear strength of joints at 220 °C for 10 min under different bonding pressures.

Figure 8 shows the shear strength of the joints under different bonding pressures. The results indicate that the shear strength of the joints increases markedly with increasing bonding pressure. The minimum shear strength was 1.5 MPa at 0 MPa, whereas the maximum reached 59.0 MPa at 10 MPa. Between 1 MPa and 5 MPa, the shear strength increased from 20.4 MPa to 40.4 MPa, corresponding to a 98.04% increase. In the range from 5 MPa to 10 MPa, the shear strength increased from 40.4 MPa to 59.0 MPa, corresponding to a 46.04% increase. Despite the continuous increase in pressure, the rate of increase in shear strength gradually diminished, particularly in the ranges 1–5 MPa and 5–10 MPa, where the growth slowed compared with the preceding interval. This indicates a diminishing marginal effect of bonding pressure on shear strength.

Microstructural analysis further corroborates this trend. At 0 MPa, extensive IMC structures were observed; however, significant gaps existed between the phases, preventing the formation of an interconnected IMC network. Large voids were also observed within the joints. As the pressure increased, the microstructure became denser, the porosity diminished, and the microstructural uniformity improved. Notably, residual Cu was scarcely detectable in joints processed at 0 MPa, whereas substantial amounts of unreacted Cu powder were visible in joints processed at 10 MPa. It is speculated that liquid Ga is extruded from the sandwich joint at higher pressures, reducing the amount of Ga available for reaction and consequently leaving more Cu unreacted.

#### 3.2.2. Effect of Bonding Time on Shear Strength and Microstructures of Joints

**Figure 9:**
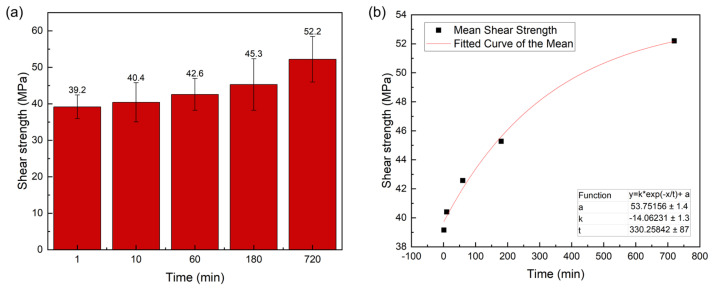
(**a**) Shear strength of joints at 220 °C and 5 MPa for different bonding times. (**b**) Fitting results for shear strength average values (the solid line represents an empirical fit to highlight the saturation tendency of shear strength with bonding time).

Figure 9a shows the shear strength of the joints at different bonding times. As the bonding time increases, the shear strength exhibits an overall increasing trend. Specifically, the shear strength reached 39.2 MPa at 1 min, 40.4 MPa at 10 min, 42.6 MPa at 60 min, 45.3 MPa at 180 min, and peaked at 52.2 MPa at 720 min. Fitting the shear strength data (see Figure 9b) indicates that the saturation trend indicates diminishing strengthening with time: the joint strength increases rapidly at early stages due to fast wetting/reaction and IMC network formation, but the improvement slows at longer times because IMC thickening and local Ga depletion make the process diffusion limited.

Microstructural analysis revealed the effect of bonding time on the joint microstructure. At the shortest bonding time (1 min), the joint primarily consisted of residual Cu powder, Ga-rich phases, CuGa_2_, and a small quantity of Cu_9_Ga_4_. As the bonding time increased, the fractions of residual Cu powder and Ga-rich phases gradually decreased. Concurrently, the CuGa_2_ phase gradually transformed into Cu_9_Ga_4_, leading to an increase in the fraction of the Cu_9_Ga_4_ phase. At 180 min, only a small amount of the initial large-particle-size Cu powder remained in the joint. At 720 min, the Cu powder was almost completely consumed and replaced by the Cu_9_Ga_4_ phase. Overall, although the fractions of the individual phases in the joint continuously evolve with time, the microstructure remains predominantly characterized by interconnected Cu–Ga IMC. Consequently, the corresponding variation in the shear strength of the joint is relatively small.

#### 3.2.3. Effect of Bonding Temperature on Shear Strength and Microstructures of Joints

**Figure 10:**
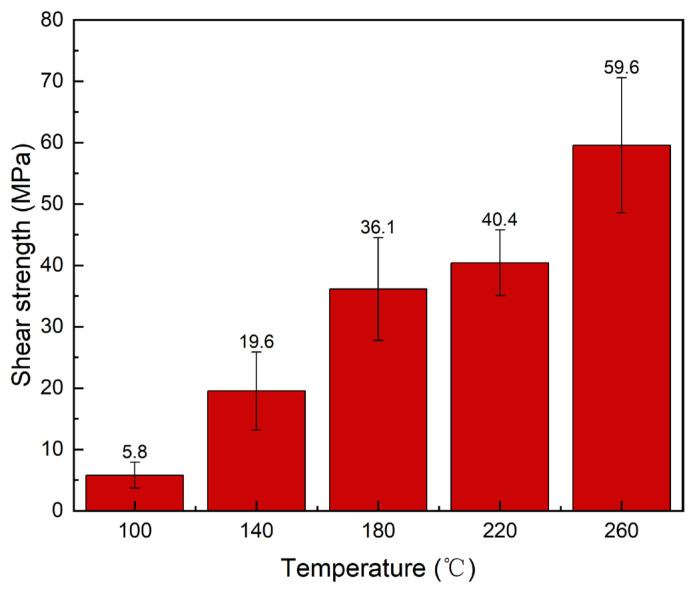
Shear strength of joints at 10 min and 5 MPa under different bonding temperatures.

Figure 10 shows the shear strength of joints at different bonding temperatures. As the temperature increased, the shear strength of joints exhibited an upward trend, though the enhancement effect of temperature on shear strength was not linear. Within the 100 °C to 140 °C temperature range, shear strength increased from 5.8 MPa to 19.6 MPa, representing a 237.93% increase. From 140 °C to 180 °C, the shear strength further increased to 36.1 MPa, representing an 84.18% increase; between 180 °C and 220 °C, the shear strength rose from 36.1 MPa to 40.4 MPa, an 11.91% increase. At 260 °C, the shear strength reached 59.6 MPa, representing a 47.52% increase. Overall, the improvement in shear strength gradually plateaued within the 100–220 °C temperature range. However, when the bonding temperature was elevated to 260 °C, the temperature-dependent enhancement effect on the shear strength of the joint resumed.

Regarding microstructure, the microstructure of the joints varied under different temperature conditions. At 100 °C, extensive CuGa_2_ formation was observed, with interconnections forming an IMC network. Nevertheless, large areas of Ga-rich phase persisted within the joint, likely contributing to its relatively low shear strength. Between 100 °C and 180 °C, the IMC primarily consisted of CuGa_2_. At 220 °C, a small amount of Cu_9_Ga_4_ phase began to appear. Within the bonding range of 100 °C to 220 °C, the proportions of residual Cu powder and Ga-rich phase in the joints gradually decreased with increasing temperature. This can be attributed to higher reaction temperatures that accelerate the reaction between Cu and Ga. However, at 260 °C, the microstructure at the joint interface exhibited distinct phenomena: the proportion of Ga-rich phase increased, the Cu_9_Ga_4_ phase formed extensively, and the Cu powder was nearly completely consumed. According to the Cu–Ga phase diagram, this may occur because the reaction temperature exceeds the melting point of CuGa_2_ (254 °C), causing a significant shift in the reaction mechanism within the joint. The CuGa_2_ formed in the joint melts and rapidly reacts with Cu particles to form Cu_9_Ga_4_, which exhibits superior mechanical properties.

#### 3.2.4. Fracture Mode of Joints

**Figure 11:**
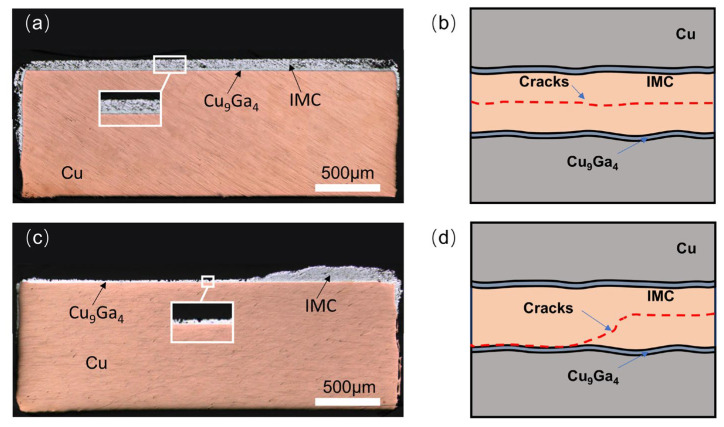
Schematic diagram of fracture interface cross-section and typical fracture surface morphology: (**a**) type I fracture joint interface; (**b**) schematic diagram of type I fracture; (**c**) type II fracture joint interface; (**d**) schematic diagram of type II fracture.

Based on the preceding shear-strength results, the fracture surfaces of the joints were examined, and the corresponding fracture modes were summarized (see Figure 11). As shown in Figure 3, a continuous and dense Cu_9_Ga_4_ phase was present at the Cu interface of the joint. In Figure 11, this phase can be clearly distinguished from the more complex IMC network within the interior of the joint. Because the Ga-rich phase inside the joint cannot provide effective mechanical support, it acts as a preferred path for crack propagation. Furthermore, according to the study of Guo et al. [47], Cu_9_Ga_4_ exhibits superior mechanical properties compared with CuGa_2_. Consequently, during shear testing, cracks initially tend to propagate along the Ga-rich phase or along voids, and ultimately fracture through the CuGa_2_ crystals.

Two primary shear-fracture modes were observed in the joints. Type I cracks: As shown in Figure 11a,b, cracks initiate within the IMC matrix and propagate along the Ga-rich phase and the CuGa_2_ phase, traversing the entire IMC interface. Type II cracks: As shown in Figure 11c,d, cracks initiate at the Cu_9_Ga_4_/IMC interface and propagate along this interface until complete joint fracture occurs, or extend along the Ga-rich phase or the CuGa_2_ phase within the joint into the IMC network until fracture. This behaviour is governed by the local distribution of phases within the joint. In addition, no delamination of the joint along the Cu/Cu_9_Ga_4_ interface was observed, indicating strong metallurgical bonding at this interface.

## 4. Conclusions

In this work, Cu substrates were successfully bonded using an in-house-formulated Cu–Ga composite paste. The microstructure, electrical conductivity, and shear strength of the joints were investigated as a function of paste composition. For the optimized composition, the effects of TLPB parameters (bonding time, temperature, and pressure) on the joint microstructure and shear strength were further examined, and the mechanisms by which these parameters influence joint performance, as well as the fracture behavior of the joints were discussed. The main conclusions can be summarized as follows:

1. The joint microstructure is mainly composed of CuGa_2_, Cu_9_Ga_4_, residual Cu, and Ga-rich regions, where CuGa_2_ and Cu_9_Ga_4_ form an interconnected network around Cu particles while a dense Cu_9_Ga_4_ layer develops on the Cu substrate.
2. By screening Cu particle size (Cu_PS_) and Cu mass fraction (Cu_MF_), the optimal paste was identified as Cu_PS_ = 10–20 μm and Cu_MF_ = 25 wt%, yielding a dense and uniform IMC framework with limited voids, an electrical conductivity of ~1.1 × 10^7^ S·m^−1^, and a shear strength of ~52.2 MPa.
3. Bonding pressure, temperature, and time markedly affect microstructural evolution and joint properties. The shear strength increases with increasing pressure, temperature, and bonding time, but the strengthening rate gradually decreases and approaches saturation. Within the investigated range, pressure and temperature dominated the strength improvement, whereas bonding time showed a comparatively weaker effect. Notably, a shear strength of 39.2 MPa was achieved at 220 °C and 5 MPa within 1 min, demonstrating the feasibility of high-strength bonding in a very short time.
4. Fracture preferentially propagates through Ga-rich/CuGa_2_ regions, whereas the dense Cu_9_Ga_4_ framework suppresses crack growth; reducing Ga-rich regions and voids improves reliability.

The Cu–Ga composite paste system proposed in this work is applicable to the fabrication of interconnect joints in power chip packaging, offering both high electrical conductivity and bonding strength. By employing a TLPB process, the realization of Cu–Ga interconnect joints within a short bonding time is made feasible, providing experimental data and theoretical support for the development of highly reliable interconnect structures for high-temperature applications.

## Figures and Tables

**Table 1:** The EDS analysis results of different points in Figure 3.

Element	Point 1	Point 2	Point 3	Point 4	Point 5	Point 6	Point 7	Point 8
Cu	99.95	57.7	34.22	56.63	33.76	60.94	0.82	9.73
Ga	0.05	42.3	65.78	43.37	66.24	39.06	99.18	90.27
Atom ratio of Cu/Ga	1999.00	1.36	0.52	1.31	0.51	1.56	0.01	0.11

## Data Availability

The original contributions presented in this study are included in the article/Supplementary Material. Further inquiries can be directed to the corresponding authors.

## Supplementary Materials

The following supporting information can be downloaded at: https://www.mdpi.com/article/10.3390/ma19020314/s1, Figure S1: (a) SEM images of the joint prepared using a Cu–Ga paste (CuPS = 30–40 μm, CuMF = 25 wt%) after reaction at 220 °C and 5 MPa for 12 h; (b) Cu/Ga elemental maps of rectangle b in (a). Figure S2: SEM image of a sandwich joint with different CuPS and CuMF at 400× magnification after reacting at 220 °C for 12 h. Figure S3: Microstructural interfaces of joints under different pressure, time, and temperature conditions.
