# Supplementary material for: Study on Microstructure and Properties of Micron Copper Powder-Liquid Metal Gallium Composite Interconnect Joint
Source: Materials (Basel). 2026 Jan 13;19(2):314. doi: 10.3390/ma19020314 (PMC12842934; doi:10.3390/ma19020314)
Supplement: Supplementary file 1 [file materials-19-00314-s001.zip › materials-4071663-supplementary.pdf]

materials

MDPI

Article

Study on Microstructure and Properties of Micron Copper Powder-Liquid Metal Gallium Composite Interconnect Joint

Bo Wang [1,2], Siliang He [2,4], Guopei Zhang [3], Menghao Liu [2*], Kaixuan He [1], Wei Huang [2] and Kailin Pan [2*]

[1] East China Institute of Photo-Electron IC, Bengbu 233000, China; wangbo_guet@163.com (B.W.); hekxsr@163.com (K.H.)
[2] Guangxi Key Laboratory of Advanced Packaging and System Integration, School of Mechanical and Electrical Engineering, Guilin University of Electronic Technology, Guilin 541004, China; siliang_he@guet.edu.cn (S.H.); huang0773@guet.edu.cn (W.H.)
[3] Goertek Inc., Weifang 261000, China; 0521825@163.com (G.Z.)
[4] Research Institute of Sun Yat-sen University in Huizhou, Huizhou, 516081, China
* Correspondence: lmenghao15@163.com (M.L.); pankl@guet.edu.cn (K.P.)

Academic Editor: Firstname Lastname

Received: date
Revised: date
Accepted: date
Published: date

Citation: To be added by editorial staff during production.

Copyright: © 2025 by the authors. Submitted for possible open access publication under the terms and conditions of the Creative Commons Attribution (CC BY) license (https://creativecommons.org/licenses/by/4.0/).

Supplementary Materials

Figure S1 displays the elemental mapping results for Cu/Ga across selected regions within Figure 3.

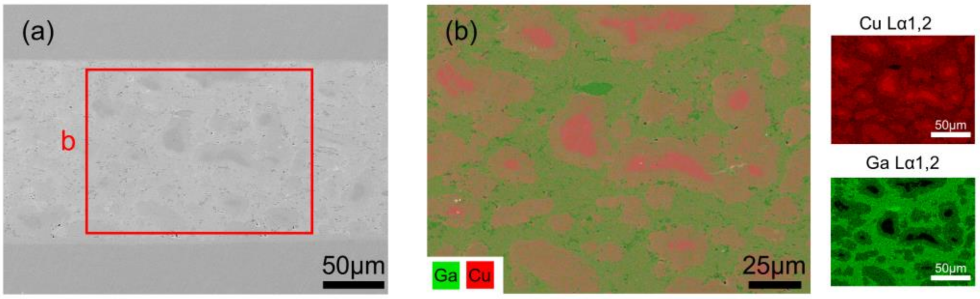

Figure S1. (a) SEM images of the joint prepared using a Cu-Ga paste (Cu_{PS} = 30-40 μm, Cu_{MF} = 25 wt%) after reaction at 220 °C and 5 MPa for 12 h; (b) Cu/Ga elemental maps of rectangle b in (a).

Figure S2 presents SEM micrographs of joints at 400× magnification under different Cu powder particle sizes (Cu_{PS}) and Cu mass fractions (Cu_{MF}) (Figure 4 in the main text corresponds to locally enlarged regions of Figure S2). These images provide a more comprehensive understanding of the overall morphology of the joints. It can be observed that the interfacial structure primarily consists of the Cu substrates on both sides and a Cu–Ga interconnected structure in the middle. Due to the highly compact microstructure in certain regions, only selected images are annotated accordingly.

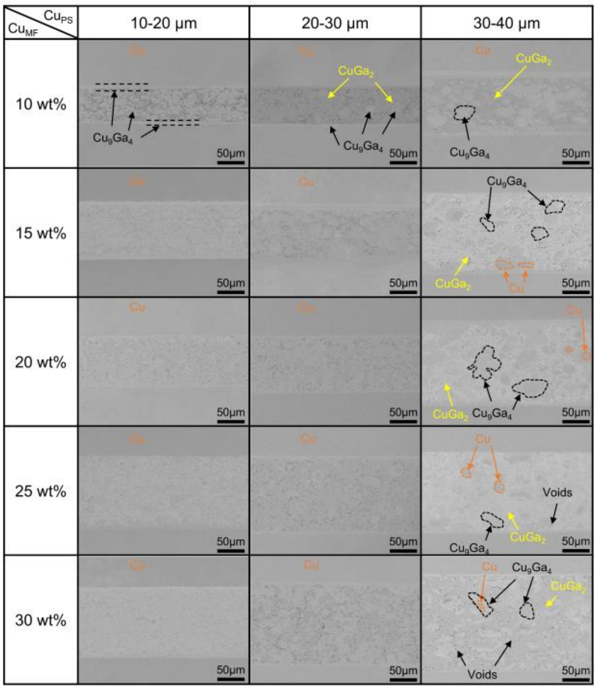

Figure S2. SEM image of a sandwich joint with different Cu_{PS} and Cu_{MF} at 400× magnification after reacting at 220 °C for 12 hours.

Figure S3 presents SEM images of interfacial microstructure of joints under various TLPB conditions (the images in Figure 7 of the main text are locally magnified regions of Figure S3). These images provide a more comprehensive understanding of the overall morphology of the joints.

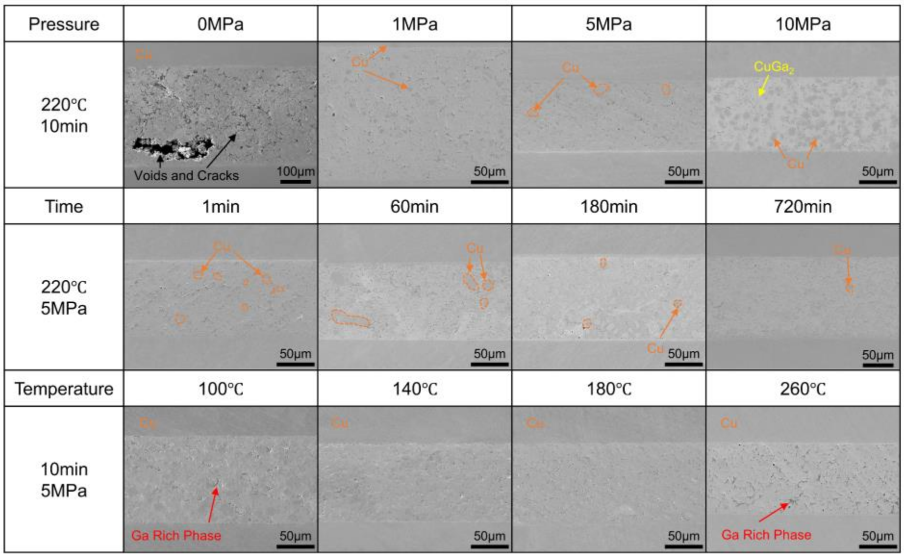

Figure S3. Microstructural interfaces of joints under different pressure, time, and temperature conditions.
